# Visual Sequelae of Computer Vision Syndrome: A Cross-Sectional Case-Control Study

**DOI:** 10.1155/2021/6630286

**Published:** 2021-04-02

**Authors:** Mohammed Iqbal, Omar Said, Ola Ibrahim, Ashraf Soliman

**Affiliations:** ^1^Department of Ophthalmology, Faculty of Medicine, Sohag University, Sohag, Egypt; ^2^Department of Ophthalmology, Faculty of Medicine, Fayoum University, Fayoum, Egypt; ^3^Department of Ophthalmology, Faculty of Medicine, Ain Shams University, Cairo, Egypt

## Abstract

**Purpose:**

To assess the visual, ocular, extraocular, and multifocal electroretinography (mfERG) outcomes of computer vision syndrome (CVS) among medical students.

**Methods:**

This study was designed as a cross-sectional case-control study that included 733 medical students. All students completed a specially designed and validated CVS questionnaire survey (CVS-F3). Students from the control (No-CVS) and CVS groups underwent comprehensive ophthalmic examinations including the mfERG examinations. Our main outcome measures included uncorrected and corrected distance visual acuity (UDVA and CDVA, resp.) measurements, subjective and cycloplegic refractions, slit-lamp examination, intraocular pressure measurement, pupillary reflexes tests, ocular movements' tests, dry eye disease tests, and fundus and mfERG examinations.

**Results:**

The CVS-F3 identified that 87.9% of students had complaints that might be related to CVS. We documented a 76% prevalence rate in students undergoing an ophthalmologic exam. The most common ocular and extraocular complaints included visual blur and headache (40.9% and 46.8%, resp.). Statistical logistic and linear regression analyses showed that refractive errors, prolonged screen-hours, close eye-screen distance, improper gaze angle, poor screen-resolution, and screen-glare were risk factors for developing CVS and influencing its severity. In the mfERG subgroup, 42.5% demonstrated reduced amplitudes of mfERG rings and quadrants, indicating reduced foveal responses.

**Conclusion:**

Surveys cannot yield an accurate CVS prevalence. However, they help to identify subjects at risk who should be comprehensively assessed to confirm or exclude CVS diagnosis. Smartphone misuse primarily caused CVS among users. Our mfERG findings might be a sign of potential CVS visual sequelae; however, future studies are warranted. Clinicians need to understand these sequelae to appropriately identify and treat CVS.

## 1. Introduction

Digital technologies are now universal and have spread worldwide; thus, digital behaviour has dramatically changed peoples' lifestyles. Previous studies have reported that individuals interact with digital screens for up to 12 hours daily [1, 2], and the American Optometric Association defined a combination of ocular and extraocular symptoms that affects the screen users as computer vision syndrome (CVS) [3]. However, our current understanding of digital technologies and their harmful impact on the eye and public health [1, 4, 5], visual performance [6–8], sleep patterns [7, 8], circadian rhythms [5, 8], musculoskeletal system activities [3, 4], and underlying physiological mechanisms [9] remains incomplete and replete with misconceptions [1]. Therefore, educational programs that include protective measures and health campaigns are necessary [10].

Computer vision syndrome (CVS) is also called by other names as digital eye strain (DES) [3], occupational asthenopia [11], digital asthenopia [1], and video display terminal syndrome (VDTS) [12, 13]. CVS ocular symptoms include visual blur with an underlying mechanism that is not fully understood [10], dry eye disease (DED) [3, 14, 15], eye redness and irritation, eyestrain, fatigue, discomfort [14, 16], difficulty in refocusing the eyes, and diplopia [3, 4]. CVS extraocular symptoms include headache; sleep disturbances; depression [7, 8]; musculoskeletal aches, such as neck/shoulder/back pain [2–17]; difficulty in writing or holding objects; and pain in thumbs, fingers, or wrists because of tendonitis and/or arthritis [18–20].

Smartphones are used extensively worldwide by people of all ages [21–23] and are characterised by a close viewing distance [24], related high-definition resolution [14], thousands of time-consuming applications and games in stores, and 24/7 Internet connectivity [2, 22]. Smartphone usage is thought to be responsible for the sharp rise in CVS prevalence and severity among users, including paediatric populations [25]. Studies have suggested that smartphones are responsible for the emergence of new, yet not understood, visual performance defects [10], smartphone-associated social and work distraction [18], and insomnia [5, 7–9, 12, 26]. There is no ideal method to document the actual prevalence of CVS worldwide. The recorded prevalence of CVS varies among different studies [4, 9, 12, 27–29], with a range from less than 10% to over 90%.

Most studies that reported a high prevalence of CVS were conducted on university students [30–37], technicians [11], bankers [38], office workers [39], government employees [40], computer users [12, 24], video gamers, visual display terminal users or workers [13, 26, 41], and children [24, 42, 43]. Most of these studies used subjective methods, primarily validated structured questionnaires [12, 29], or symptom scales [28, 35].

No study in the literature investigated macular integrity or function in CVS [26]; however, one study suggested that smartphones are responsible for new and unexplained visual performance defects, including CVS-associated visual blur [10]. Meanwhile, another study reported visual sensitivity reduction following smartphone use in the dark [44].

In this study, our primary goal was to document the potential visual and ocular sequelae among medical students using a subjective CVS questionnaire and a complete ophthalmic examination. Furthermore, our secondary goal was to calculate the CVS prevalence within the study cohort. Finally, we sought to document the correlation between the subjective survey and objective clinical findings.

## 2. Materials and Methods

This study obtained the approval of the Institutional Review Board (IRB) in the Faculty of Medicine, Sohag University, Egypt. The trial registry number was obtained from ClinicalTrials.gov (registry number: NCT04398212). This study was conducted in accordance with the tenets of the Declaration of Helsinki. Our study protocol included subjective information (i.e., the CVS questionnaire) and an objective ophthalmic examination of medical students in three Egyptian Universities. Prior to study enrolment, informed consent was obtained from these students after having explained to them the nature and possible consequences of the study.

### 2.1. Sample Size

Using an alpha level of 0.01 and the survey sample size determination table created by Bartlett et al. [45], we determined that the minimum sample size required for this study was 623 participants.

### 2.2. Subjective Self-Assessment Evaluation

#### 2.2.1. Computer Vision Syndrome: Form 1

In 2017, our research team developed and piloted a well-structured CVS questionnaire form (CVS-F1). It consisted of 20 questions that detected CVS prevalence among medical students at Sohag University. Our main objective at this point was to assess the reliability and validity of the CVS-F1. After our study was published in January 2018 [32], other researchers from different nations outside Egypt requested to use our CVS-F1 in their surveys and studies in 2018 and 2019. Results from these studies further confirmed the reliability and validity of the CVS-F1.

#### 2.2.2. Computer Vision Syndrome: Form 3

In this study, we modified the CVS-F1 by adding more questions on the environmental, screen-use habits, and associated screen factors. The new modified questionnaire was identified as CVS-F3 (28 questions, S1 appendix in Supplementary Materials), which we used in this study. Our main aim by using CVS-F3 was to document whether the users' habits and screen-styles were affecting the number and frequency of CVS complaints and its role in preventing the development of CVS. This modification minimised standard errors in the new version of the questionnaire (CVS-F3) and ensured it was more specific.

Our study included 733 medical students who were randomly assigned to complete the CVS-F3 regardless of their grade or age. The potential complaints and consequences of CVS were carefully explained to all participants before they responded to CVS-F3. The 733 surveyed students were classified into two groups according to their final diagnosis following their ophthalmic examination. The two groups consisted of the CVS group, which included students who were diagnosed with CVS, and the No-CVS group (control group), which included students who were not diagnosed with CVS. We also defined an mfERG subgroup, which included a random sample of students from both the CVS and control groups who underwent an mfERG examination.

In our study, the CVS final diagnosis was documented based on four major criteria. The first criterion was the presence of one or more ocular complaints related to the time of screen-use. The second criterion was the presence of one or more extraocular complaints related to the time of screen-use. The third criterion was the presence of one or more complaint-attacks every month over the last 12 months. The fourth criterion was ophthalmic examinations documenting DED, conjuctival hyperemia, reduced visual acuity, associated refractive errors, and/or mfERG abnormalities.

For greater clarity, we assessed all students prior to their grouping. Based on their examination and final diagnosis, we managed to certify the students who had CVS and identified them as the CVS group. On one hand, the remaining students who did not have CVS were identified as the control group (No-CVS group). Thereafter, we created an additional subgroup named the mfERG subgroup. Our main aim was to randomly assign an equal and small number of the students from both the CVS and control groups, using STATA software program, version 14.2, as a sample to undergo mfERG assessments to minimise the expenses of the costly mfERG examinations. Therefore, the mfERG subgroup (*n* = 40 students) was actually a mixed subgroup that included 20 students each from both the CVS and control groups. Furthermore, within the mfERG subgroup, we examined only one eye from each student (40 eyes of 40 mfERG-students) to minimise any potential statistical bias if both eyes of the same subject were included in the data statistical analysis. Therefore, we performed a coin toss, and only the left eyes of the 40 mfERG-students were included in the mfERG examinations.

All students underwent a complete clinical ophthalmic assessment and evaluation at the Ophthalmology Examination Unit in the Department of Ophthalmology. All students underwent visual acuity assessments, which included an uncorrected distance visual acuity (UDVA) measurement, corrected distance visual acuity (CDVA) measurement, subjective refraction, cycloplegic refraction, testing ocular movements, slit-lamp examination, intraocular pressure measurement, pupillary reflexes, and fundus examination. All examined students also underwent DED testing, which included the tear film break-up time test (TBUT) and the Schirmer test. The exclusion criteria included amblyopia; strabismus; anisometropia; CDVA worse than 0.00 logMAR; refractive errors higher than 6 D myopia, 4 D hyperopia, or 4 D astigmatism; difference between subjective and cycloplegic refraction >1 D; near vision abnormalities; and previous or current systemic or eye disease or surgery. The excluded students were not included in this study.

To perform the Schirmer test, we temporally inserted a Schirmer strip (Schirmer Ophthalmic Strip; Surgi Edge) into the lower fornix. Eyes were gently closed for 5 minutes, after which the amount of wetting was measured. If it was <10 mm, we considered it abnormal. To perform the TBUT, we inserted a fluorescein strip (1 mg fluorescein sodium I.P.; Surgi Edge, Ahmedabad, Gujarat, India) into the lower fornix, and the students were requested to blink several times. The students then underwent a slit-lamp examination with the cobalt blue-filter; if black holes were found in the tear film in less than 10 seconds, it was considered abnormal.

A random subsample of 40 eyes of 40 students from both groups was examined with the mfERG device (RETIscan; Roland Instruments, Wiesbaden, Germany) in accordance with the standard protocol for mfERG of the International Society for Clinical Electrophysiology of Vision (ISCEV). The mfERG stimulus used was 61 hexagons in dilated subjects with system age-matched norms. The protocol adhered to ISCEV standards.

### 2.3. Statistical Analysis

Stata statistical software (version 14.2; StataCorp LP, College Station, TX, USA) was used for data analysis. The mean, standard deviation, range, and median values were used to describe the quantitative data. Numbers and percentages were used to describe the qualitative data. The chi-square test or Fisher's exact test were used for comparisons between categorical variables. The Mann–Whitney *U* test was used for comparisons between two groups, and the Kruskal–Wallis test was used for comparisons between three or more groups because the variables were not normally distributed. A binary logistic regression analysis was used to determine the factors that affected the occurrence of CVS, whereas a linear regression analysis was used to determine the factors that affected the number of CVS symptoms. Excel or STATA was used to produce the relevant graphs. A *P* value of <0.05 was considered statistically significant.

## 3. Results

This study included 733 medical students (305 males [41.6%] and 428 females [58.4%]) with a mean age of 21.8 ± 1.5 years. The survey group (*n* = 733) was subdivided into the control group (*n* = 176; 24%) and the CVS group (*n* = 557; 76%) based on ophthalmic examination.

### 3.1. CVS-F3 Outcomes

CVS-F3 documented that 87.9% of the surveyed students had one or more ocular and/or extraocular complaints. However, only 70.8% of them reported that these complaints were associated with their screen use, that is, during or immediately after screen-use. In short, we will address here the most relevant CVS-F3 statistical analysis outcomes.

The most common ocular symptom included blurred vision in 40.9% of students, while the most common extraocular symptom was headache (46.8%). All ocular and extraocular complaints worsened with prolonged screen-hours, except for depression (*P*=0.2). Student complaints became worse with prolonged screen-hours at night than during the daytime. All ocular symptoms became worse with an increase in the number of screen-years, except for eye strain and redness (*P*=0.10 and 0.49, resp.). Sleep disturbance (insomnia) was the only extraocular symptom that worsened with the number of screen-years (*P*=0.001). In addition, we recorded no statistically significant differences between the level of screen brightness or screen-mode (i.e., interrupted or continuous screen-hours) and any of the student's complaints (*P*=0.6 and 0.14, resp.).

Our survey outcomes revealed that the most common screen used by students was a smartphone. In addition, 504 students (68.8%) used various types and systems of smartphones. Specifically, 397 students (54.2%) used Android smartphones, 97 students (13.2%) used iOS smartphones (i.e., iPhones), and 10 students (1.4%) used other smartphone brands (Table 1). Laptops were the second most common screen used by the students, reported by 129 users (17.6%). All ocular and extraocular symptoms were significantly higher in students who used smartphones compared with those students who used laptops and desktop monitors, with the exception of double vision, depression, and inability to hold objects (*P*=0.41, 0.80, and 0.35, resp., Table 1). We found statistically significant differences between devices with which desktop computer users had the least risk of developing CVS complaints (Table 1). Finally, our CVS-F3 demonstrated that there was no statistically significant difference between Android and Apple smartphone users regarding the mean CVS number of symptoms (*P*=0.36).

The most common associated screen behaviour CVS factors recorded by our CVS-F3 were a close eye-screen distance (42.6% of surveyed students), watching the screen in the dark (33.7%), improper gaze angle as the screen edge was at/above horizontal eye level (28.2%), texting with both thumbs (28.8%), small font size (23.9%), and poor or improper lighting conditions (20.9%).

Regarding the frequency and severity of CVS complaints, 75% of total surveyed students reported that their complaints were frequently in the form of symptoms-attacks (repeated complaints on a monthly basis); however, only 70.8% of students stated that their symptoms-attacks were directly related to screen use, that is, typically during or immediately after their screen-use. On the other hand, 3.8% of students reported that their symptom-attacks were not related to screen use, that is, mostly not in the form of CVS symptoms. CVS-F3 recorded that the mean number of symptom-attacks/month was 3.6 ± 2.9 (ranging from 0 to 15 attacks/month). In contrast, the mean number of years that subjects had these symptom-attacks was 3.6 ± 2.9 (ranging from 0 to 8 years). Therefore, CVS may be responsible for chronic complaints in some cases.

Our CVS-F3 outcomes revealed that refractive errors represented a major CVS factor associated with CVS occurrence and the number of symptoms. In our sample, 56.5% of students had refractive errors and showed statistically significantly higher percentages of most of CVS ocular and extraocular symptoms.

We discovered statistically significant differences between students who were texting with both thumbs (*n* = 211) and the students who are not texting with both thumbs (*n* = 522) regarding joint pains in wrists and fingers, inability to hold objects well, and difficulty writing with a pen as these extraocular symptoms were worse with texting with both thumbs (all *P* < 0.0001). These findings suggest that texting with both thumbs represented the mean causative screen-related CVS factor linked to the development of joint pains in wrists and fingers, with an inability to hold objects well, and difficulty in writing with a pen.

Tables 2–4 summarise the CVS-F3 univariate, multivariate, and final multivariate logistic regression analyses' factors that affect the occurrence of CVS. Tables 5 and 6 and appendices S2-S3 in Supplementary Materials summarise the CVS-F3 multivariate and final multivariate logistic regression analyses' factors that affect the occurrence of blurred vision and dry eyes, respectively. Tables 7 and 8 summarise the multivariate and final multivariate linear regression analyses' factors affecting the total number of symptoms.

### 3.2. Ophthalmologic Examination Outcomes

Table 9 represents the comparative outcomes between the control and CVS groups. The mean sphere, cylinder, and SE were significantly higher in the CVS than in the control group (*P* < 0.0001). These findings suggest that refractive errors, myopia, and astigmatism were risk factors associated with CVS occurrence. Both UDVA and CDVA were significantly better in the control than in the CVS group (*P* < 0.0001). Both TBUT and Schirmer test showed significantly reduced means in the CVS than in the control group (*P* < 0.0001, Table 9).

### 3.3. mfERG Examination Outcomes

**Figure 1:**
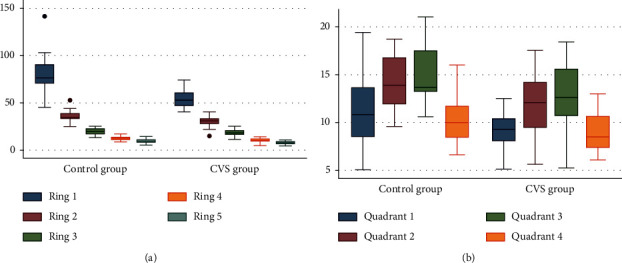
mfERG subgroup outcomes. (a) Plot showing the main differences in mfERG Rings P1 amplitudes between the control and the CVS groups. (b) Plot showing the main differences in mfERG Quadrants P1 amplitudes between the control and the CVS groups.

The mfERG subgroup included 40 eyes of 40 students (16 males and 24 females). The mfERG examination took nearly 15–20 minutes to be completed. In the mfERG analysis, the normal mfERG ranges were determined internal to the system.  Table 10 and Figure 1 summarise the data summary of the mfERG subgroup.

In the control group, all 20 eyes exhibited a normal mfERG examination with normal foveal responses, including a preserved foveal peak (first positive peak, P1), and the amplitude density (AD) was within the normal range, demonstrating normal foveal function. In the CVS group, only three eyes exhibited a normal foveal response, while the remaining 17 eyes exhibited a reduced foveal response and were identified as positive cases.

**Figure 2:**
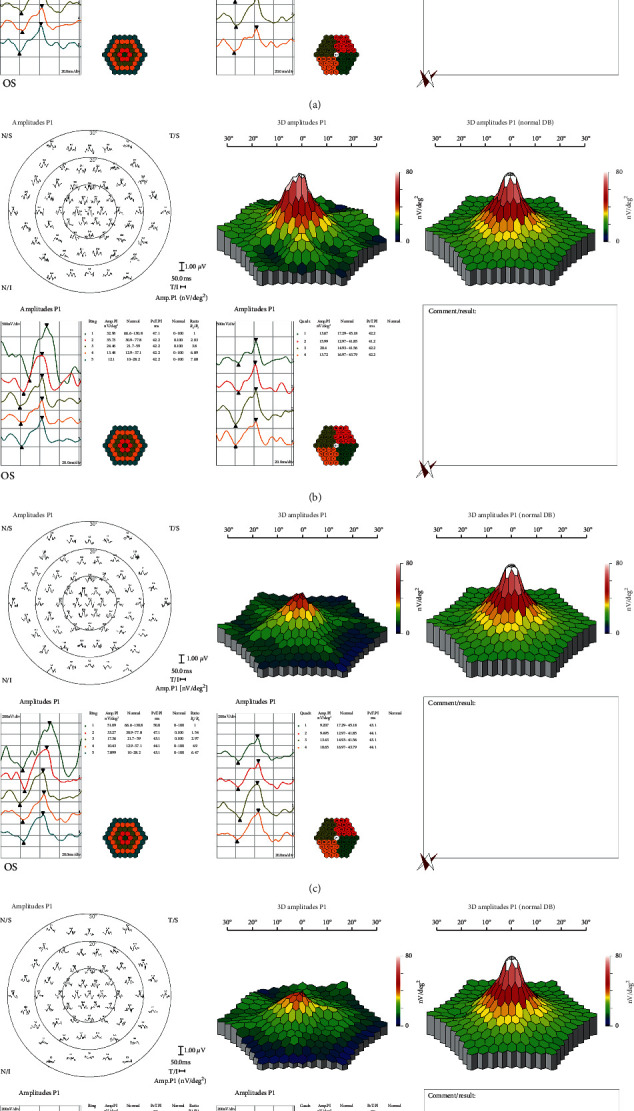
Multifocal electroretinography outcomes: (a) and (b) two students from the control group with normal foveal responses; (c) and (d) two students from the CVS group with reduced foveal responses.

In these 17 positive cases, the P1 AD for Ring 1 was 51.53 ± 7.24 nV/deg2 (mean ± SD). The parafoveal and perifoveal rings also showed a significant reduction as Rings 3, 4, and 5 showed P1 AD of 18.87 ± 3.85, 10.72 ± 2.64, and 8.02 ± 2.07 nV/deg2, respectively. These findings reveal foveal dysfunction and may explain the reduction in CDVA.  Figure 2 demonstrates examples of mfERG findings in two students from the CVS group.

We found that the students who were spending more than 3 screen-hours daily (*P*=0.006), spending most of their screen-time at night (*P*= 0.03), and adjusting their screen-illumination >50% (*P*=0.01) had potentially higher likelihood of developing mfERG changes (Table 10).

## 4. Discussion

The main purpose of this study was to document the CVS-associated visual or ocular sequelae. In addition, we also sought, as a secondary purpose, to detect the actual CVS prevalence among the medical students included in our study sample size. There were three primary outcomes in our study. First, we demonstrated that comprehensive ophthalmic examinations and investigations were more accurate than subjective questionnaires regarding the diagnosis of the actual CVS-related sequelae, severity, and prevalence. Second, our findings revealed that the misuse of smartphones is mainly responsible for the increase in CVS prevalence and severity. Third, our mfERG findings might be a sign of potential CVS visual sequelae in high-risk CVS subjects, which could be confirmed or denied in future studies.

Although CVS-F3 outcomes reported potential CVS-related symptoms in 87.9% of surveyed students, ophthalmic examinations documented only a 76% CVS prevalence rate among the assessed students. Therefore, we think that subjective questionnaires might be overestimating the actual prevalence rates of CVS, while comprehensive ophthalmic examinations are more accurate in documenting actual CVS prevalence rates. The relatively better statistical outcomes of a desktop computer in comparison with smartphones and laptops can be explained based on the larger screen size, further screen distance, low cost, and the fact that these are neither easily portable nor handheld screens.

We believe that the smartphone itself might not be the underlying cause for exacerbating CVS but the way of its usage by the subjects might be the problem. Our conclusion was based on the outcomes of the final logistic regression analysis which found that improper close eye-screen viewing distance, improper gaze angle, poor screen design, poor screen-resolution, incorrect seating posture, texting with both thumbs, and associated refractive errors represented the main risk factors of CVS occurrence (*P* < 0.0001). Our final linear regression analysis exhibited that the latter factors together with poor lighting conditions, small screen size, and small font size were the key factors impacting the number associated with CVS symptoms and complaints (*P* < 0.0001). Visual blur caused by CVS was mainly triggered by a close eye-screen distance, improper gaze angle, screen-glare, poor lighting conditions, and associated refractive errors. All aforementioned risk factors may be cofounders in the context of our hypothesis that stipulated that the further the screen was from the eye, the less severe the CVS; indeed, we documented eyestrain in 19% of desktop computer users versus 50% of smartphone users.

Prolonged and continuous screen-hours require the bilateral use of both sets of intraocular and extraocular muscles (e.g., ciliary, constrictive pupillae, and medial recti muscles) to adjust the focus and achieve the best visual performance. Poor eye coordination or inadequate eye focusing might be caused by improper or extremely close viewing distance, improper screen brightness, poor screen-resolution, screen-glare in old screens, and/or uncorrected refractive errors, which finally increase CVS severity. The small screen size and small font size also increase eye strain and fatigue due to inadequate eye focusing. We observed that the distance between the screen and the user's eyes decreases as the screen size decreases and the severity of CVS increases. Therefore, the greatest CVS severity was associated with misuse of smartphones, whereas the lowest severity was associated with desktop computers.

Similar to our outcomes, Golebiowski et al. [46] concluded that the underlying aetiological mechanisms aggravating smartphone-related CVS severity differ from those associated with desktop computers. Long et al. [24] reported that eye strain increases as the viewing distance between a smartphone and the eye decreases. The CVS-F3 revealed that there were 4.1 ± 2.8 CVS smartphone-associated symptoms (range: 0–13) with four symptoms on average. Meanwhile, Kim et al. [47] reported a higher rate of smartphone-associated symptoms (5–7 CVS symptoms) in adolescents. This may be related to a variety of cofactors, such as screen size, poor screen-resolution, and old or new versions of smartphones.

Comparisons between the CVS-F3 outcomes and other studies' surveys have revealed major differences that could be attributed to our larger sample size, behavioural practices, and different study demographics. Mowatt et al. [33] studied the relationship between ergonomic activities and CVS in 409 university students. They reported higher percentages of complaints than CVS-F3 regarding eye strain, DED, neck pain, blurred vision, and double vision. Pulla et al. [31] found that 75.1% of 300 engineering students used many digital gadgets, and the CVS prevalence was 60.3%. Dessie et al. [40] reported 69.5% CVS prevalence with a higher prevalence of the most common CVS complaints, including blurred vision, eye strain, and eye irritation. Logaraj et al. [34] compared CVS prevalence between medical students and engineering students and reported that the CVS prevalence was higher among engineering students (81.9%) than among medical students (78.6%), similar to our results (76%). Consistent with our results, these studies found that, as the number of screen-hours increased, the severity of CVS symptoms also increased.

Al Rashidi and Alhumaidan [37] reported that 77.8% of their students were myopes, and the remaining students were emmetropes. In addition, they revealed that the presentation of CVS was more severe in myopic students who used contact lenses compared with those wearing spectacles. Finally, they concluded that there was a statistically significant relationship between myopia and the severity of CVS (*P* < 0.001). Similarly, we also recorded significantly higher refractive errors in the CVS group than the control group with a significant relationship between myopia, contact lens wearing, and CVS severity. Billones et al. [16] concluded that to minimise CVS eye strain, the best distance associated with the least eye saccades and strain was 50–62.5 cm. Similarly, we found that eye strain decreased as the distance between the user and the screen increased. In this study, eye strain occurred in only 19% of desktop computer users versus up to 50% of smartphone users. We concluded that the further the screen was from the eye, the less severe the CVS was.

Our mfERG findings pointed to macular cone/bipolar cell dysfunction. We posit that CVS might have elicited these recorded mfERG changes in this small subsample of students from light exposure as a result of cone adaptation, electrode/focusing effects, or the spectral output of the devices which varied between subjects. Exposure to high levels of longer wavelength light could adapt L and M cones better than a shorter wavelength exposure. However, we remain unsure of whether or not these students were using colour adjustments to their smartphones or study displays, as we did not investigate this point.

The main limitation of our study consisted in our inability to perform mfERG on many participants due to costs. mfERG is also difficult and time-consuming; therefore, it might not be suitable for all study participants. Another limitation to our study included the subjects' tendency to use multiple screens, which made it difficult to isolate a particular effect for a particular screen. Finally, we do acknowledge that our study was not a population-based study. Therefore, our recorded CVS prevalence rate can be only applied to the medical students' category in our region, but not to the population in our country.

## 5. Conclusions

In conclusion, subjective questionnaire surveys alone, without an ophthalmic examination, are not ideal for documenting true CVS prevalence. CVS can be only accurately diagnosed with comprehensive ophthalmic examinations and investigations. The misuse of smartphones, regardless of the manufacturer, was the main aetiological agent responsible for the development and sequelae of CVS. Our study showed that CVS might have caused mfERG changes with reduced foveal responses; however, this potential screen-induced foveal dysfunction and its impact on visual acuity need to be confirmed in future studies.

## Figures and Tables

**Table 1:** Relationship between symptoms and type of commonest/primary screen used.

	Apple *N* = 97	Android *N* = 397	Laptop *N* = 129	Desktop computer *N* = 95	iPad/Tab *N* = 5	Other screens *N* = 10	*P* value
*Ocular symptoms*							
Blurred vision	47.4%	47.6%	41.1%	15.8%	0	40.0%	<0.0001^*∗*^
Dry eyes	24.7%	26.5%	20.9%	10.5%	20.0%	30.0%	0.02^*∗*^
Eye strain/fatigue	43.3%	50.1%	41.1%	19.0%	40.0%	60.0%	<0.0001^*∗*^
Eye redness	17.5%	24.7%	24.0%	8.4%	0	40.0%	0.002^*∗*^
Double vision	2.1%	3.0%	0.8%	0	0	0	0.41^*∗*^
Refocusing difficulties	20.6%	20.7%	17.8%	6.3%	0	10%	0.02^*∗*^
Near vision difficulties	13.4%	16.1%	15.5%	4.2%	0	10%	0.04^*∗*^
Unclear objects	44.33%	45.1%	38.0%	16.8%	0	70.0%	<0.0001^*∗*^

*Extraocular symptoms*							
Headache	44.3%	53.9%	45.0%	20.0%	40.0%	70.0%	<0.0001^*∗*^
Insomnia	22.7%	21.7%	18.6%	5.3%	20.0%	10.0%	0.003^*∗*^
Depression	1.0%	0.8%	0	0	0	0	0.80^*∗*^
Neck pains	45.4%	49.9%	38.0%	22.1%	40.0%	40.0%	<0.0001^*∗*^
Joint pains	35.1%	36.3%	18.6%	10.5%	0	30.0%	<0.0001^*∗*^
Inability to hold objects	3.1%	5.0%	3.1%	1.1%	0	10.0%	0.35^*∗*^
Difficulty to write	13.4%	6.3%	2.3%	5.3%	0	10.0%	0.03^*∗*^

*Number of symptoms*							
Mean ± SD	3.8 ± 2.9	4.1 ± 2.8	3.2 ± 3.0	1.5 ± 2.4	1.6 ± 1.8	4.3 ± 1.8	0.0001
Median (range)	4 (0 : 13)	4 (0 : 12)	3 (0 : 11)	0 (0 : 10)	1 (0 : 4)	3.5 (2 : 7)	
CVS diagnosed with ophthalmic examination	77 (79.4%)	340 (85.6%)	92 (71.3%)	36 (37.9%)	2 (40.0%)	10 (100%)	<0.0001

^*∗*^Fisher's exact test was used as there are more than 20% of cells containing expected value less than 5.

**Table 2:** Univariate logistic regression analysis model of factors affecting the occurrence of computer vision syndrome.

Variable	No CSV *N* = 176	CSV *N* = 557	Odds ratio (95% confidence interval)	*P* value
Age/years	21.4 ± 1.9	22.0 ± 1.4	1.3 (1.2 : 1.4)	<0.0001
Gender				
Males	88 (50.0%)	217 (39.0%)	1	
Females	88 (50.0%)	340 (61.0%)	1.6 (1.1 : 2.2)	0.01
Total daily screen-hours	3.8 ± 1.2	5.3 ± 1.9	1.7 (1.5 : 2.0)	<0.0001
Screen-years	4.1 ± 1.7	4.7 ± 1.9	1.2 (1.1 : 1.3)	<0.0001
Screen-time				
Day	64 (36.4%)	176 (31.6%)	1	
Night	112 (63.6%)	381 (68.4%)	1.2 (0.9 : 1.8)	0.24
Screen-mode				
Interrupted	127 (72.2%)	452 (81.2%)	1	
Continued	49 (27.8%)	105 (18.9%)	0.6 (0.4 : 0.9)	0.01
Commonest used screen				
Desktop computer screen	59 (33.5%)	36 (6.5%)	1	
Apple smartphone	20 (11.4%)	77 (13.8%)	6.3 (3.3 : 12.0)	<0.0001
Android smartphone	57 (32.4%)	340 (61.0%)	9.8 (5.9 : 16.1)	<0.0001
Laptop	37 (21.0%)	92 (16.5%)	4.1 (2.3 : 7.2)	<0.0001
iPad/tablet/other screens	3 (1.7%)	12 (2.2%)	6.5 (1.7 : 24.8)	0.01
Screen size				
Large	93 (52.8%)	258 (46.3%)	1	
Medium/small	83 (47.2%)	299 (53.7%)	1.3 (0.9 : 1.8)	0.13
Screen-version				
New	158 (89.8%)	453 (81.3%)	1	
Old	18 (10.2%)	104 (18.7%)	2.0 (1.2 : 3.4)	0.01
Screen brightness (%)	43.3 ± 23.3	39.2 ± 24.5	0.99 (0.98 : 1.00)	0.054
Study medicine using				
Books	33 (18.8%)	82 (14.7%)	1	
Screens/both	143 (81.2%)	475 (85.3%)	1.3 (0.9 : 2.1)	0.20
Main screen-time purpose is				
Medicine	112 (63.6%)	249 (44.7%)	1 2.2 (1.5 : 3.1)	
Social	64 (36.4%)	308 (55.3%)		<0.0001
Previous DED diagnosis	14 (8.0%)	84 (15.1%)	2.1 (1.1 : 3.7)	0.02
Refractive errors/wearing	82 (46.6%)	332 (59.6%)	1.7 (1.2 : 2.4)	0.003
Contact lenses wearer	14 (8.0%)	29 (5.2%)	0.6 (0.3 : 1.2)	0.18
Poor lighting conditions	12 (6.8%)	141 (25.3%)	4.6 (2.5 : 8.6)	<0.0001
Watch screen in the dark	36 (20.5%)	211 (37.9%)	2.4 (1.6 : 3.6)	<0.0001
Upper screen edge at/above horizontal eye level	2 (1.1%)	205 (36.8%)	50.0 (12.4 : 206.4)	<0.0001
Close eye-screen distance	14 (8.0%)	298 (53.5%)	13.3 (7.5 : 23.6)	<0.0001
Uncomfortable seating postures	2 (1.1%)	77 (13.8%)	14.0 (3.4 : 57.4)	<0.0001
Texting with both thumbs	10 (5.7%)	201 (36.1%)	9.4 (4.8 : 18.2)	<0.0001
Screen-glare	2 (1.1%)	43 (7.7%)	7.3 (1.7 : 30.4)	0.01
Poor screen-resolution or design	2 (1.1%)	49 (8.8%)	8.4 (2.0 : 34.9)	0.003
Small font size	21 (11.9%)	154 (27.7%)	2.8 (1.7 : 4.6)	<0.0001

**Table 3:** Multivariate logistic regression analysis model of factors affecting the occurrence of computer vision syndrome.

Variable	Odds ratio (95% confidence interval)	*P* value
Gender		
Males	1	
Females	1.8 (1.0 : 3.2)	0.047
Total daily screen-hours	2.1 (1.7 : 2.6)	<0.0001
Commonest used screen		
Desktop computer screen	1	
Apple smartphone	1.0 (0.3 : 3.3)	0.94
Android smartphone	3.2 (1.2 : 8.1)	0.02
Laptop	1.7 (0.7 : 4.8)	0.23
iPad/tablet/other screens	3.8 (0.4 : 40.5)	0.27
Screen size		
Large	1	
Medium/small	1.7 (0.96 : 2.9)	0.07
Screen-version		
New	1	
Old	0.8 (0.3 : 2.2)	0.63
Main screen-time purpose is		
Medicine	1	
Social	1.44 (0.7 : 3.0)	0.33
Previous DED diagnosis	3.8 (0.9 : 15.7)	0.06
Refractive errors/wearing	2.1 (1.2 : 3.8)	0.01
Contact lenses wearer	0.03 (0.005 : 0.1)	<0.0001
Poor lighting conditions	1.46 (0.6 : 3.7)	0.42
Watch screen in the dark	0.9 (0.5 : 1.8)	0.75
Upper screen edge at/above horizontal eye level	47.5 (10.1 : 225.1)	<0.0001
Close eye-screen distance	11.2 (5.1 : 24.6)	<0.0001
Uncomfortable seating postures	13.4 (2.4 : 75.5)	0.003
Texting with both thumbs	7.6 (3.1 : 18.6)	<0.0001
Screen-glare	2.0 (0.3 : 15.5)	0.52
Poor screen-resolution or design	35.8 (4.4 : 295.1)	0.001
Small font size	1.9 (0.88 : 4.1)	0.11

**Table 4:** Final multivariate logistic regression analysis model of factors affecting the occurrence of computer vision syndrome.

Variable	Odds ratio (95% confidence interval)	*P* value
Total daily screen-hours	2.0 (1.7 : 2.5)	<0.0001
Commonest used screen		
Desktop computer screen	1	
Apple smartphone	1.5 (0.6 : 3.9)	0.43
Android smartphone	4.6 (2.24 : 9.6)	<0.0001
Laptop	1.8 (0.8 : 4.2)	0.17
iPad/tablet/other screens	4.3 (0.6 : 31.0)	0.15
Previous DED diagnosis	5.2 (1.4 : 19.0)	0.01
Refractive errors/wearing spectacles	2.3 (1.4 : 4.0)	0.002
Contact lenses wearer	0.03 (0.01 : 0.1)	<0.0001
Upper screen edge at/above horizontal eye level	44.3 (10.0 : 196.5)	<0.0001
Close eye-screen distance	10.8 (5.2 : 22.5)	<0.0001
Uncomfortable seating postures	19.1 (3.7 : 98.5)	<0.0001
Texting with both thumbs	6.1 (2.7 : 14.2)	<0.0001
Poor screen-resolution or design	48.2 (6.6 : 354.9)	<0.0001

**Table 5:** Multivariate logistic regression analysis of factors affecting the occurrence of blurred vision.

Variable	Odds ratio (95% confidence interval)	*P* value
Age/years	1.1 (0.99 : 1.3)	0.06
Gender		
Males	1	
Females	1.2 (0.8 : 1.7)	0.38
Total daily screen-hours	1.0 (0.9 : 1.2)	0.37
Screen-years	0.98 (0.88 : 1.1)	0.63
Commonest used screen		
Desktop computer screen	1	
Apple smartphone	2.4 (1.0 : 5.5)	0.04
Android smartphone	2.0 (0.95 : 4.2)	0.07
Laptop	2.1 (0.99 : 4.5)	0.054
iPad/tablet/other screens	0.9 (0.2 : 3.9)	0.88
Screen brightness (%)	1.00 (0.99 : 1.0)	0.44
Study medicine using		
Books	1	
Screens/both	1.6 (0.97 : 2.7)	0.07
Main screen-time purpose is		
Medicine	1	
Social	1.3 (0.8 : 1.9)	0.30
Previous DED diagnosis	2.1 (1.2 : 3.8)	0.01
Refractive errors/wearing	2.0 (1.4 : 2.8)	<0.0001
Contact lenses wearer	0.7 (0.3 : 1.6)	0.41
Poor lighting conditions	2.6 (1.7 : 4.1)	<0.0001
Watch screen in the dark	1.0 (0.68 : 1.6)	0.87
Upper screen edge at/above horizontal eye level	2.0 (1.3 : 2.9)	0.001
Close eye-screen distance	2.1 (1.5 : 3.0)	<0.0001
Uncomfortable seating postures	1.3 (0.7 : 2.3)	0.38
Texting with both thumbs	1.7 (1.2 : 2.5)	0.004
Screen-glare	2.8 (1.2 : 6.5)	0.02
Poor screen-resolution or design	1.9 (0.8 : 4.4)	0.13
Small font size	1.5 (0.97 : 2.3)	0.07

**Table 6:** Final multivariate logistic regression analysis of factors affecting the occurrence of blurred vision.

Variable	Odds ratio (95% confidence interval)	*P* value
Age/years	1.1 (1.0 : 1.3)	0.02
Commonest used screen		
Desktop computer screen	1	
Apple smartphone	2.7 (1.2 : 5.7)	0.01
Android smartphone	2.5 (1.3 : 4.7)	0.01
Laptop	2.2 (1.1 : 4.6)	0.03
iPad/tablet/other screens	1.0 (0.3 : 4.1)	0.97
Previous DED diagnosis	2.1 (1.3 : 3.6)	0.004
Refractive errors/wearing	1.9 (1.3 : 2.7)	<0.0001
Poor lighting conditions	2.6 (1.7 : 4.0)	<0.0001
Upper screen edge at/above horizontal eye level	2.0 (1.4 : 3.0)	<0.0001
Close eye-screen distance	2.2 (1.5 : 3.0)	<0.0001
Screen-glare	3.0 (1.5 : 6.2)	0.003

**Table 7:** Multivariate linear regression analysis of factors affecting the total number of symptoms.

Variable	Regression coefficient (95% confidence interval)	*P* value
Age/years	0.1 (−0.04 : 0.2)	0.23
Gender		
Males	1	
Females	0.5 (0.2 : 0.8)	0.001
Total daily screen-hours	0.2 (0.1 : 0.3)	<0.0001
Screen-years	0.03 (−0.1 : 0.1)	0.53
Commonest used screen		
Desktop computer screen	1	
Apple smartphone	0.3 (−0.3 : 0.9)	0.36
Android smartphone	0.4 (−0.2 : 0.9)	0.19
Laptop	0.2 (−0.3 : 0.8)	0.39
iPad/tablet/other screens	0.4 (−0.7 : 1.5)	0.50
Screen size		
Large	1	
Medium/small	0.3 (0.04 : 0.6)	0.02
Study medicine using		
Books	1	
Screens/both	0.5 (0.1 : 0.9)	0.03
Main screen-time purpose is		
Medicine	1	
Social	0.2 (−0.1 : 0.6)	0.24
Previous DED diagnosis	0.9 (0.5 : 1.4)	<0.0001
Refractive errors/wearing	0.6 (0.3 : 0.9)	<0.0001
Contact lenses wearer	0.2 (−0.5 : 0.9)	0.53
Poor lighting conditions	1.3 (0.9 : 1.7)	<0.0001
Watch screen in the dark	0.0003 (−0.3 : 0.3)	0.998
Upper screen edge at/above horizontal eye level	1.5 (1.1 : 1.8)	<0.0001
Close eye-screen distance	1.2 (0.9 : 1.6)	<0.0001
Uncomfortable seating postures	1.2 (0.7 : 1.7)	<0.0001
Texting with both thumbs	1.7 (1.3 : 2.00)	<0.0001
Screen-glare	1.6 (0.9 : 2.3)	<0.0001
Poor screen-resolution or design	1.6 (0.9 : 2.3)	<0.0001
Small font size	0.7 (0.4 : 1.1)	<0.0001

**Table 8:** Final multivariate linear regression analysis of factors affecting the total number of symptoms.

Variable	Regression coefficient (95% confidence interval)	*P* value
Gender		
Males	1	
Females	0.6 (0.3 : 0.9)	<0.0001
Total daily screen-hours	0.2 (0.1 : 0.3)	<0.0001
Screen size		
Large	1	
Medium/small	0.3 (0.05 : 0.6)	0.02
Previous DED diagnosis	1.1 (0.:1.5)	<0.0001
Refractive errors/wearing	0.7 (0.4 : 1.0)	<0.0001
Poor lighting conditions	1.3 (1.0 : 1.7)	<0.0001
Upper screen edge at/above horizontal eye level	1.5 (1.2 : 1.8)	<0.0001
Close eye-screen distance	1.3 (1.00 : 1.6)	<0.0001
Uncomfortable seating postures	1.3 (0.8 : 1.7)	<0.0001
Texting with both thumbs	1.8 (1.4 : 2.1)	<0.0001
Screen-glare	1.4 (0.8 : 2.0)	<0.0001
Poor screen-resolution or design	1.4 (0.9 : 2.0)	<0.0001
Small font size	0.8 (0.4 : 1.1)	<0.0001

**Table 9:** Differences between the control and CVS groups.

Parameters	Control group (*n* = 176 eyes of 176 students) (Mean ± SD) Median (range)	CVS group (*n* = 557 eyes of 557 students) (Mean ± SD) Median (range)	Mean difference (Control-CVS) 95% confidence of Interval	*P* value
Visual outcomes (logMAR):				
UDVA	0.13 ± 0.12	0.31 ± 0.25	−0.18	<0.0001
	0.1 (−0.1 : 0.5)	0.3 (−0.1 : 1.1)	(−0.22 : −0.09)	
CDVA	−0.016 ± 0.04	−0.002 ± 0.01	−0.014	<0.0001
	0 (−0.1 : 0)	0 (−0.1 : 0)	(−0.018 : −0.008)	
Subjective refraction (D):				
Sphere	−0.51 ± 1.13	−0.90 ± 1.12	0.39	<0.0001
	−0.13 (−4:2.5)	−0.75 (−5:4)	(−0.02 : 0.53)	
Cylinder	−0.25 ± 0.61	−0.51 ± 0.74	0.26	<0.0001
	0 (−4:1)	−0.25 (−4:2.75)	(0.17 : 0.41)	
SE	−0.63 ± 1.19	−1.16 ± 1.42	0.53	<0.0001
	−0.5 (−4:2.5)	−0.88 (−6.25 : 4.25)	(0.24 : 0.68)	

DED tests:				
Tear film break-up time:				<0.0001
TBUT in seconds	12.38 ± 1.78	8.93 ± 2.16	3.45	
	12 (7 : 17)	9 (2 : 15)	(4.26 : 3.12)	
Abnormal TBUT test (<10 seconds)	5 eyes (2.8%)	336 eyes (60.3%)		<0.0001
Schirmer test: Schirmer test in mm	19.57 ± 4.07	10.68 ± 4.37	8.89	<0.0001
	20 (8 : 29)	9 (5 : 26)	(10.94 : 8.46)	
Abnormal Schirmer test (<10 mm)	5 eyes (2.8%)	301 eyes (54%)		<0.0001
Slit-lamp examination:				
Conjunctival hyperemia (eye redness)	11 eyes (6.3%)	181 eyes (32.5%)		<0.0001
Watery/mucous discharge	0 eyes (0%)	3 eyes (0.5%)		<0.0001
Normal fundus examination:	100%	100%		
Students/eyes documented with				
Diagnosed CVS cases	0 cases (0%)	557 cases (100%)		<0.0001
Reduced UDVA (worse than 0.00 logMAR)	101 eyes (57.4%)	409 eyes (73.4%)		0.001
Reduced CDVA (worse than 0.00 logMAR)	0 eyes (0%)	0 eyes (0%)		1.00
Diagnosed DED cases	5 eyes (2.8%)	336 eyes (60.3%)		<0.0001

UDVA: uncorrected distance visual acuity; CDVA: corrected distance visual acuity; TBUT: tear film break-up time test; CVS: computer vision syndrome; DED: dry eye disease; SE: spherical equivalent; logMAR logarithm of the minimum angle of resolution.

**Table 10:** Data summary of the mfERG subgroup.

Parameters	mfERG students from control group (*n* = 20 eyes of 20 students) (Mean ± SD) Median (range)	mfERG of students from CVS group (*n* = 20 eyes of 20 students) (Mean ± SD) Median (range)	Mean difference (mini−control−risk) 95% confidence of interval	*P* value
Age	22.35 ± 1.53	21.95 ± 2.14	0.4	0.50
	22 (19 : 25)	21 (19 : 25)	(−0.79 : 1.59)	
Gender:				
Male	8 (40%)	8 (40%)		1.00
Female	12 (60%)	12 (60%)		
Screen-hours:				
1 h	4 (20%)	0		
2 h	3 (15%)	1 (5%)		0.09
3 h	3 (15%)	1 (5%)		
4 h	2 (10%)	4 (20%)		
5 h	5 (25%)	5 (25%)		
≥6 h	3 (15%)	9 (45%)		
- mfERG students < 3 screen-hours	10 (50%)	2 (10%)		0.006
- mfERG students ≥3 screen-hours	10 (50%)	18 (90%)		
Screen-time:				
Day	12 (60%)	5 (25%)		0.03
Night	8 (40%)	15 (75%)		
Screen-illumination:				
10%	5 (25%)	1 (5%)		0.06
30%	7 (35%)	3 (15%)		
50%	5 (25%)	5 (25%)		
80%	2 (10%)	6 (30%)		
100%	1 (5%)	5 (25%)		
- mfERG students ≤50% illumination	17 (85%)	9 (45%)		0.01
- mfERG students >50% illumination	3 (15%)	11 (55%)		
Commonest screen used:				
Desktop computers	0	2 (10%)		0.4
Laptops	1 (5%)	2 (10%)		
Apple	4 (20%)	4 (20%)		
Android	14 (70%)	12 (60%)		
Other screens	1 (5%)	0		
mfERG findings:				
I-amplitudes P1 (nV/deg2):				
Ring 1 (normal 66.6–130.8)	80.60 ± 19.71	54.01 ± 9.17	26.59 (16.75 : 36.43)	<0.0001
	76.49 (45.26 : 141.4)	52.86 (40.43 : 74.09)		
Ring 2 (normal 30.9–77.8)	35.75 ± 6.94	30.55 ± 5.97	5.20 (1.05 : 9.34)	0.02
	34.76 (25.08 : 52.74)	30.97 (15.17 : 40.61)		
Ring 3 (normal 21.7–59)	19.80 ± 3.80	18.57 ± 3.67	1.22 (−1.17 : 3.62)	0.31
	20.08 (13.38 : 25.3)	18.27 (11.54 : 25.36)		
Ring 4 (normal 12.9–37.1)	12.62 ± 2.52	10.50 ± 2.40	2.11 (0.54 : 3.69)	0.01
	12.87 (8.87 : 17.38)	10.21 (4.98 : 14.37)		
Ring 5 (normal 10–28.2)	9.69 ± 2.55	7.82 ± 1.81	1.88 (0.46 : 3.29)	0.01
	9.13 (5.51 : 14.51)	7.74 (4.57 : 10.93)		
II-amplitudes P1 (nV/deg2):				
Quadrant 1 (normal 15.8–42.74)	11.26 ± 3.76	9.17 ± 2.01	2.09 (0.16 : 4.03)	0.03
	10.83 (5.05 : 19.41)	9.29 (5.12 : 12.48)		
Quadrant 2 (normal 15.98–42.75)	14.39 ± 2.81	12.03 ± 2.98	2.36 (0.51 : 4.22)	0.01
	13.91 (9.58 : 18.71)	12.09 (5.63 : 17.54)		
Quadrant 3 (normal 15.18–42.05)	15.13 ± 3.06	12.80 ± 3.46	2.33 (0.24 : 4.42)	0.03
	13.69 (10.6 : 21.02)	12.60 (5.23 : 18.4)		
Quadrant 4 (normal 13.87–39.61)	10.27 ± 2.41	9.20 ± 2.18	1.07 (−0.40 : 2.54)	0.15
	9.98 (6.62 : 16.02)	8.49 (6.09 : 16.02)		
III-foveal functions:				
Normal foveal response (21 eyes)	20 eyes (100%)	3 eyes (15%)		<0.0001
Reduced foveal response (19 eyes)	0 (0%)	17 eyes (85%)		

## Data Availability

Patients' data used to support the findings of this study are available from the corresponding author upon request.

## Supplementary Materials

Supplementary Materials S1 appendix: CVS-F3. S2 appendix: multivariate logistic regression analysis of factors affecting the occurrence of dry eye. S3 appendix: final multivariate logistic regression analysis of factors the affecting occurrence of dry eye. S4 appendix: univariate linear regression analysis of factors affecting the total number of symptoms.
