# Supplementary material for: Visual Sequelae of Computer Vision Syndrome: A Cross-Sectional Case-Control Study
Source: J Ophthalmol. 2021 Apr 2;2021:6630286. doi: 10.1155/2021/6630286 (PMC8035040; doi:10.1155/2021/6630286)
Supplement: Supplementary Materials — S1 appendix: CVS-F3. S2 appendix: multivariate logistic regression analysis of factors affecting the occurrence of dry eye. S3 appendix: final multivariate logistic regression analysis of factors the affecting occurrence of dry eye. S4 appendix: univariate linear regression analysis of factors affecting the total number of symptoms. [file 6630286.f1.zip › 6630286.f1/S1 Appendix. CVS-F3.docx]

**Computer Vision Syndrome Survey Form 3 (CVS-F3)**

**Please mark your answers (√): (University) Date: Name:**

- **Age: 16 17 18 19 20 21 22 23 24 25 26**
- **Gender: Male Female**
- **How many hours do you spend on your digital screen every 24 hours (total screen-hours)?**

**0 1 2 3 4 5 6 7 8 9 10 11 12 13 14 15 16**

- **How many of your total screen-hours do you spend on your digital screen during the daytime?**

**0 1 2 3 4 5 6 7 8 9 10 11 12**

- **How many of your total screen-hours do you spend on your digital screen at night?**

**0 1 2 3 4 5 6 7 8 9 10 11 12**

- **How many years have you spent using screens in this manner?**

**0 1 2 3 4 5 6 7 8 9 10 11 12 13 14 15 16**

- **Do you spend most of your screen-hours during the day or at night? Day Night**
- **The hours you spend on your digital screen are? Continuous Interrupted**
- **What are the digital screens you commonly use? (Please select one or more answers):**

**Desktop computer Laptop iPad/Tab Apple smartphone Android smartphone others**

- **What is the most common primary/single screen you use? (Please select one answer only):**

**Desktop computer Laptop iPad/Tab Apple smartphone Android smartphone others**

- **What is the screen-size of the most common individual/single screen you use?**

**Small-sized screen Medium-sized screen Large-sized screen**

- **What is the screen-version of the most common individual/single screen you use?**

**New-version screen (within last 2 years) Old-version screen**

- **To what average level do you illuminate your primary screen (i.e., screen-brightness) in the dark?**

**10% 20% 30% 40% 50% 60% 70% 80% 90% 100%**

- **Do you have any of the following symptoms frequently with screen use over last 12 months?**

**(Please select all answers that apply; if none apply, leave blank):**

***Ocular symptoms:***

| **Blurred**[**vision**](https://en.wikipedia.org/wiki/Visual_perception) |  | [**Eye strain**](https://en.wikipedia.org/wiki/Asthenopia) **and fatigue** |  | **Difficulty in refocusing the eyes** |  |
| --- | --- | --- | --- | --- | --- |
| [**Dry eyes**](https://en.wikipedia.org/wiki/Dry_eyes) |  | **Eye redness and irritation** |  | **Near vision discomfort/difficulty** |  |
|  |  | **Double vision/diplopia** |  | **Unclear objects post-screen use** |  |

***Extraocular symptoms:***

| [**Headache**](https://en.wikipedia.org/wiki/Headache) |  | **Insomnia** |  | **Neck/shoulder/back pain** |  | **Inability to hold objects well** |  |
| --- | --- | --- | --- | --- | --- | --- | --- |
|  |  | **Depression** |  | **Joint pain in fingers and wrists** |  | **Difficulty to write using a pen** |  |

- **How many symptoms-attacks on average, if any, you suffer from every month over last 12 months?**

**0 1 2 3 4 5 6 7 8 9 10 11 12 13 14 15 16**

- **How many years, on average, do you suffer from these symptoms-attacks, if any?**

**0 1 2 3 4 5 6 7 8 9 10 11 12 13 14 15 16**

- **Are your symptoms-attacks associated with screen use? N/A Yes No**
- **Do you have previous diagnosis of dry eye disease or use eye drops to treat it? Yes No**
- **Do you have any refractive error or wearing glasses? Yes No**
- **Do you wear contact lenses or have contact lenses related diseases? Yes No**
- **Do you have previous eye or systemic disease or surgery? Yes No**
- **Do you feel that digital screens affect your lifestyle and eye health? Yes No**
- **Are you willing to decrease your screen hours to guard against CVS? Yes No**
- **Is your medical school involved in mandated computer system use program? Yes No**
- **How do you usually study medicine? Screens alone Books alone Both**
- **What is the main screen you usually use to study medicine?**

**Desktop computer Laptop iPad/Tab Apple smartphone Android smartphone Others None**

- **What is your main purpose that consumes most of your screen-time? (Select one answer only):**

**Medicine/Science Social communication/Entertainment others**

- **Do you have any of the following practices frequently with screen use? (Please select all answers that apply; if none apply, leave blank):**

| **Poor screen- resolution or design** |  | **Screen- glare** |  | **Poor lighting conditions** |  |
| --- | --- | --- | --- | --- | --- |
| **Screen edge at/above horizontal eye level** |  | **Close eye-screen distance** |  | **Watch screen in the dark** |  |
| **Uncomfortable seating postures** |  | **Small-font size** |  | **Texting with both thumbs** |  |

**Consent: By completing this survey, I agree that the data or outcomes of CVS-F3 and/or ophthalmic examination will be used as a part of CVS research project for publication worldwide. Yes No**
