# Supplementary material for: Visual Sequelae of Computer Vision Syndrome: A Cross-Sectional Case-Control Study
Source: J Ophthalmol. 2021 Apr 2;2021:6630286. doi: 10.1155/2021/6630286 (PMC8035040; doi:10.1155/2021/6630286)
Supplement: Supplementary Materials — S1 appendix: CVS-F3. S2 appendix: multivariate logistic regression analysis of factors affecting the occurrence of dry eye. S3 appendix: final multivariate logistic regression analysis of factors the affecting occurrence of dry eye. S4 appendix: univariate linear regression analysis of factors affecting the total number of symptoms. [file 6630286.f1.zip › 6630286.f1/S2 Appendix. Multivariate logistic regression analysis of factors affecting the occurrence of dry eye.docx]

**S2 Appendix.** Multivariate logistic regression analysis of factors affecting the occurrence of dry eye

| **Variable** | **Odds ratio (95% confidence interval)** | **P value** |
| --- | --- | --- |
| **Age/years** | 1.0 (0.8:1.2) | 0.84 |
| **Gender**  Males  Females | 1  1.4 (0.8:2.3) | 0.23 |
| Total daily screen-hours | 1.1 (0.9:1.2) | 0.28 |
| Screen-years | 1.0 (09:1.2) | 0.78 |
| **Screen-time**  Day  Night | 1  0.8 (0.5:1.4) | 0.45 |
| **Screen-mode**  Interrupted  Continued | 1  1.1 (0.6:2.0) | 0.74 |
| **Commonest used screen**  Desktop Computer Screen  Apple smartphone  Android smartphone  Laptop  iPad/Table/ Other screen | 1  1.1 (0.4:3.4)  1.1 (0.4:2.9)  1.4 (0.5:3.8)  2.1 (0.4:10.4) | 0.86  0.83  0.47  0.37 |
| **Screen size**  Large  Medium/ Small | 1  1.4 (0.9:2.3) | 0.14 |
| **Screen-version**  New  Old | 1  1.0 (43:2.4) | 0.97 |
| **Screen brightness (%)** | 0.99 (0.99:1.00) | 0.46 |
| **Study medicine using:**  Books  Screens/both | 1  1.1 (0.6:2.4) | 0.72 |
| **Main screen-time purpose is:**  Medicine  Social | 1  1.2 (0.7:2.1) | 0.57 |
| Previous DED diagnosis | 26.8 (13.4:53.7) | <0.0001 |
| Refractive errors/wearing | 1.5 (0.9:2.5) | 0.09 |
| Contact lenses wearer | 5.2 (1.5:17.5) | 0.008 |
| Poor lightening conditions | 1.7 (1.0:3.0) | 0.047 |
| Watch screen in the dark | 1.0 (0.6:1.8) | 0.90 |
| Upper screen edge at/above horizontal eye level | 1.4 (0.9:2.4) | 0.16 |
| Close eye-screen distance | 2.2 (1.3:3.7) | 0.002 |
| Uncomfortable seating postures | 1.3 (0.6:2.8) | 0.48 |
| Texting with both thumbs | 1.1 (0.7:1.88) | 0.59 |
| Screen-glare | 2.8 (1.1:6.9) | 0.03 |
| Poor screen- resolution or design | 2.5 (0.96:6.3) | 0.06 |
| Small font-size | 1.2 (0.7:2.0) | 0.57 |
