# Supplementary material for: Visual Sequelae of Computer Vision Syndrome: A Cross-Sectional Case-Control Study
Source: J Ophthalmol. 2021 Apr 2;2021:6630286. doi: 10.1155/2021/6630286 (PMC8035040; doi:10.1155/2021/6630286)
Supplement: Supplementary Materials — S1 appendix: CVS-F3. S2 appendix: multivariate logistic regression analysis of factors affecting the occurrence of dry eye. S3 appendix: final multivariate logistic regression analysis of factors the affecting occurrence of dry eye. S4 appendix: univariate linear regression analysis of factors affecting the total number of symptoms. [file 6630286.f1.zip › 6630286.f1/S3 Appendix. Final multivariate logistic regression analysis of factors the affecting occurrence of dry eye.docx]

**S3 Appendix.** Final multivariate logistic regression analysis of factors the affecting occurrence of dry eye

| **Variable** | **Odds ratio (95% confidence interval)** | **P value** |
| --- | --- | --- |
| Previous DED diagnosis | 30.0 (15.3:58.8) | <0.0001 |
| Contact lenses wearer | 5.3 (1.7:16.7) | 0.004 |
| Poor lightening conditions | 1.8 (1.1:2.9) | 0.02 |
| Close eye-screen distance | 2.6 (1.6:4.0) | <0.0001 |
| Screen-glare | 2.8 (1.3:6.0) | 0.007 |
| Poor screen- resolution or design | 2.4 (1.2:5.2) | 0.02 |
