# Supplementary material for: Visual Sequelae of Computer Vision Syndrome: A Cross-Sectional Case-Control Study
Source: J Ophthalmol. 2021 Apr 2;2021:6630286. doi: 10.1155/2021/6630286 (PMC8035040; doi:10.1155/2021/6630286)
Supplement: Supplementary Materials — S1 appendix: CVS-F3. S2 appendix: multivariate logistic regression analysis of factors affecting the occurrence of dry eye. S3 appendix: final multivariate logistic regression analysis of factors the affecting occurrence of dry eye. S4 appendix: univariate linear regression analysis of factors affecting the total number of symptoms. [file 6630286.f1.zip › 6630286.f1/S4 Appendix. Univariate linear regression analysis of factors the affecting total number of symptoms.docx]

**S4 Appendix.** Univariate linear regression analysis of factors the affecting total number of symptoms

| **Variable** | **Regression coefficient (95% confidence interval)** | **P value** |
| --- | --- | --- |
| **Age/years** | 0.3 (0.1:0.4) | <0.0001 |
| **Gender**  Males  Females | 1  0.98 (0.6:1.4) | <0.0001 |
| Total daily screen-hours | 0.5 (0.3:0.6) | <0.0001 |
| Screen-years | 0.2 (0.1:0.3) | <0.0001 |
| **Screen-time**  Day  Night | 1  0.3 (-0.2:0.7) | 0.21 |
| **Screen-mode**  Interrupted  Continued | 1  -0.3 (-0.8:0.3) | 0.31 |
| **Commonest used screen**  Desktop Computer Screen  Apple smartphone  Android smartphone  Laptop  iPad/Table/ Other screen | 1  2.3 (1.5:3.1)  2.6 (2.0:3.3)  1.8 (1.1:2.5)  1.9 (0.4:3.5) | <0.0001  <0.0001  <0.0001  0.01 |
| **Screen size**  Large  Medium/ Small | 1  0.5 (0.1:0.9) | 0.03 |
| **Screen-version**  New  Old | 1  1.1 (0.6:1.7) | <0.0001 |
| **Screen brightness (%)** | -0.01 (-0.02:-0.00003) | 0.049 |
| **Study medicine using:**  Books  Screens/both | 1  1.0 (0.4:1.6) | 0.001 |
| **Main screen-time purpose is:**  Medicine  Social | 1  0.6 (0.2:1.0) | 0.004 |
| Previous DED diagnosis | 2.7 (1.7:2.9) | <0.0001 |
| Refractive errors/wearing | 1.1 (0.7:1.5) | <0.0001 |
| Contact lenses wearer | 2.0 (1.2:2.9) | <0.0001 |
| Poor lightening conditions | 2.4 (1.9:2.9) | <0.0001 |
| Watch screen in the dark | 1.0 (0.5:1.4) | <0.0001 |
| Upper screen edge at/above horizontal eye level | 2.5 (2.1:3.0) | <0.0001 |
| Close eye-screen distance | 2.5 (2.1:2.9) | <0.0001 |
| Uncomfortable seating postures | 2.1 (1.4:2.8) | <0.0001 |
| Texting with both thumbs | 2.7 (2.3:3.2) | <0.0001 |
| Screen-glare | 2.9 (2.0:3.7) | <0.0001 |
| Poor screen- resolution or design | 2.2 (1.4:3.0) | <0.0001 |
| Small font-size | 1.5 (1.0:2.0) | <0.0001 |
